# Assessing Electronic Cigarette-Related Tweets for Sentiment and Content Using Supervised Machine Learning

**DOI:** 10.2196/jmir.4392

**Published:** 2015-08-25

**Authors:** Heather Cole-Lewis, Arun Varghese, Amy Sanders, Mary Schwarz, Jillian Pugatch, Erik Augustson

**Affiliations:** ^1^ ICF International Rockville, MD United States; ^2^ National Cancer Institute Tobacco Control Research Branch Bethesda, MD United States

**Keywords:** social media, Twitter, e-cigarette, machine learning

## Abstract

**Background:**

Electronic cigarettes (e-cigarettes) continue to be a growing topic among social media users, especially on Twitter. The ability to analyze conversations about e-cigarettes in real-time can provide important insight into trends in the public’s knowledge, attitudes, and beliefs surrounding e-cigarettes, and subsequently guide public health interventions.

**Objective:**

Our aim was to establish a supervised machine learning algorithm to build predictive classification models that assess Twitter data for a range of factors related to e-cigarettes.

**Methods:**

Manual content analysis was conducted for 17,098 tweets. These tweets were coded for five categories: e-cigarette relevance, sentiment, user description, genre, and theme. Machine learning classification models were then built for each of these five categories, and word groupings (n-grams) were used to define the feature space for each classifier.

**Results:**

Predictive performance scores for classification models indicated that the models correctly labeled the tweets with the appropriate variables between 68.40% and 99.34% of the time, and the percentage of maximum possible improvement over a random baseline that was achieved by the classification models ranged from 41.59% to 80.62%. Classifiers with the highest performance scores that also achieved the highest percentage of the maximum possible improvement over a random baseline were Policy/Government (performance: 0.94; % improvement: 80.62%), Relevance (performance: 0.94; % improvement: 75.26%), Ad or Promotion (performance: 0.89; % improvement: 72.69%), and Marketing (performance: 0.91; % improvement: 72.56%). The most appropriate word-grouping unit (n-gram) was 1 for the majority of classifiers. Performance continued to marginally increase with the size of the training dataset of manually annotated data, but eventually leveled off. Even at low dataset sizes of 4000 observations, performance characteristics were fairly sound.

**Conclusions:**

Social media outlets like Twitter can uncover real-time snapshots of personal sentiment, knowledge, attitudes, and behavior that are not as accessible, at this scale, through any other offline platform. Using the vast data available through social media presents an opportunity for social science and public health methodologies to utilize computational methodologies to enhance and extend research and practice. This study was successful in automating a complex five-category manual content analysis of e-cigarette-related content on Twitter using machine learning techniques. The study details machine learning model specifications that provided the best accuracy for data related to e-cigarettes, as well as a replicable methodology to allow extension of these methods to additional topics.

## Introduction

As evidenced by the announcement of “vape” as the Oxford dictionary word of the year in 2014, electronic cigarettes (e-cigarettes) are relevant and of interest to the general public [1]. Although the topic is pervasive to the general public, there is no definitive scientific evidence on the safety or effectiveness of e-cigarettes [2]. Furthermore, there is limited evidence on public knowledge, attitudes, and behaviors related to e-cigarettes [3-5]. This information is critical to guiding the development of public health communication, policies, and interventions regarding e-cigarettes.

With 74% of online adults using some form of social media [6], the digital landscape is continuing to evolve, and social media platforms such as Twitter have become platforms for public discourse at a local and global level on a variety of topics and events, including health and politics [7]. These discussions generate a massive amount of data that represent unfiltered public opinion and provide a unique opportunity for social science and public health research, especially for rapidly evolving topics, such as e-cigarettes.

Behavioral science and public health researchers traditionally turn to surveys, focus groups, and in-depth interviews to explore a particular topic. However, these techniques often require considerable resources, such as time and money. Additionally, these methodologies are subject to biases related to querying a person in a research setting that can affect the validity of findings (eg, social desirability bias). Furthermore, the landscape on some topics evolves rapidly and thus requires research that can be conducted quickly and with minimal resources in order to ensure that public health efforts are abreast of public knowledge, attitudes, and behaviors. Twitter analysis is currently a very active research area that offers an abundance of data for the behavioral and social sciences. The examination of this data can uncover trends in knowledge, attitudes, and behavior; inform public health and public policy; and pave the way for interventions delivered via social media, especially in the case of tobacco use and cessation [8-12]. The breadth of social media data available allows researchers to circumvent the aforementioned issues and explore opportunities to analyze this data, thus giving rise to infodemiology and infoveillance—the analysis of Internet content and electronic data sources to identify health-related trends and disease outbreaks [13]. This field may also be referred to as digital epidemiology or digital disease detection [14].

In 2009, Google Flu Trends aggregated search engine queries to track influenza activity in the United States, which strongly correlated with official surveillance data from the Influenza-like Illness Surveillance Network [15]. Google Flu Trends was able to detect regional outbreaks before conventional Centers for Disease Control and Prevention surveillance systems, bringing infoveillance and infodemiology closer to true real-time public health surveillance [16]. However, the methodology used was subject to biases and its influenza incidence predictions have often fallen short of reality [17]. Similarly, Chew and Eysenbach used infodemiology methods to monitor Twitter trends surrounding H1N1 in 2009, which included the adoption of the WHO-recommended term “H1N1” over “swine flu”, the use of retweets to disseminate information (versus opinions and personal experiences), and an early attempt at implementing automated analysis to monitor and analyze tweets in real-time [18]. Paul and Dredze [19] found that Twitter data correlate with public health metrics and knowledge such as syndromic surveillance to identify flu outbreaks, sentinel surveillance to identify the correlation of geographic behavioral risk factors and disease such as tobacco use and cancer, and the combination of both types of surveillance to identify seasonal allergies by geographic region, which lends further support for the use of social media data as cheaper and faster to obtain in comparison to survey data. Infodemiology via Twitter has also been used in studies tracking sentiment and informedness during natural disasters, misuse of antibiotics, and other public health issues and patterns [12,20-22].

Despite the widespread application of Twitter data for infodemiology, skeptics warn that the signal-to-noise ratios from sources like Twitter are very low and the demographics represented on Twitter represent younger voices with a larger proportion of minorities, making Twitter results less representative of the general public [23]. Nonetheless, as this area of research and application advances, there is increasing attention paid to the ethical challenges that arise from use of publicly available data and how to conduct research that acknowledges and addresses those challenges [14]. For example, Yin et al [24] developed a method to detect whether tweets originate from accounts run by individuals or accounts run by companies or organizations, thus providing opportunities to improve the signal-to-noise ratio when using Twitter data for infodemiology and infoveillance.

Given the growing opportunities for infodemiology in public health, it is important to continue improving upon existing methodologies for behavioral science and public health in order to increase accuracy and efficiency, as well as determining how best to utilize computational techniques to support traditional public health methods. Recent studies have implemented manual content analyses of data obtained via Twitter in order to assess public sentiment on the emerging topic of e-cigarettes [25] and the extent of e-cigarette marketing via Twitter [26]. Building on this, our research team developed a five-category coding scheme to classify and identify trends in public conversations about e-cigarettes based on information culled from Twitter over a 1-year period.

This research extended the science on previous manual content analyses for e-cigarettes because the five categories included in the manual content analysis were crafted specifically to inform public health communication, intervention, and research by focusing not only on sentiment about e-cigarettes and content of messages, but also important details such as characterization of the speaker. Findings from the manual content analysis and subsequent correlational analyses study revealed trends in e-cigarette conversations via Twitter. Results showed not only sentiment of tweets, but also the type of Twitter user discussing various categories of content, and how the conversation (and types of Twitter users driving that conversation) shifted over time. For instance, advertising and promotion-related tweets were the single largest content theme category, followed by policy-related and then health-related tweets. Additionally, everyday users of Twitter generated a greater percentage of marketing-related tweets than retailers or tobacco companies, and everyday users of Twitter were also the top producers of tweets demonstrating first-person use or intent to use e-cigarettes with e-cigarette use.

While findings from this study are informative, the e-cigarette landscape continues to grow and thus, the manual content analysis would need to be replicated over time in order to continue to discover trends. Manual annotation of data requires considerable resources and time [27]. There is an opportunity to utilize computational methods such as machine learning to enhance and extend traditional public health methods. However, it is critical to explore the best computational models to support the task of automating the classification of Twitter data.

The purpose of this study was to determine feasibility of using computational natural language processing-based supervised machine learning techniques to replicate findings of a five-category manual content analysis of Twitter data related to e-cigarettes by using the manually coded data as a training set to train machine learning algorithms. This research builds on that of the tri-axial coding scheme used by Myslín et al [25]; however, the current study is unique in that it uses a more complex five-category coding scheme. Findings from the current study stand to provide insight into specific methodological considerations (eg, type of classification algorithm, size of word grouping analysis unit, and amount of information necessary) that enhance the performance of computational models designed to identify specifics of e-cigarette-related content on Twitter such as relevance, type of Twitter user, sentiment towards e-cigarettes, and more. Study findings stand to inform the development of public health-related infodemiology tools that may be deployed retrospectively and in real-time to explore public opinion on rapidly developing topics such as e-cigarettes.

## Methods

### Overview

In this study, supervised machine learning was used to build predictive classification models that assess Twitter data for a range of e-cigarette–related factors. Multiple classification models were created that varied by underlying machine learning classification technique and word-grouping units (ie, n-grams). Performance of classification models was assessed using 10-fold cross validation. Additionally, adequacy of sample size for manually coded content was determined by plotting model performance against varying sample sizes to build learning curves.

### Data Collection and Manual Annotation

The corpus of tweets that formed the basis of this analysis was acquired from Gnip, a provider of historical Twitter data. Strategic keywords were used to collect historic tweets potentially related to e-cigarettes between May 1, 2013, and May 1, 2014. Keywords were selected by building on keyword lists used for similar research in the literature and adapted based on information of interest for the purposes of this study (Multimedia Appendix 1) [25]. Gnip provided all tweets meeting the keyword search during the time frame, which yielded 3.7 million tweets. Manual content analysis was conducted for a randomly selected subset of tweets, thus creating the dataset to be used in this analysis. Tweets were coded according to a codebook developed based on previous literature and adapted for the purposes of this study [12,18,25]. Six analysts independently coded a subset of 250 of the same tweets until an acceptable interrater reliability score was reached for each of the five categories detailed in Table 1. Interrater reliability was determined using the Fleiss’ kappa statistic and a score of at least 0.64 was obtained for each category, indicating substantial or good agreement [28,29]. Definitions of the categories (relevance, sentiment, user description, genre, theme) can be found in Table 1 and Multimedia Appendix 2.

A total of 17,098 tweets were coded for relevance, of which 10,128 (59.23%) were found to be relevant and interpretable and therefore coded for the additional categories of sentiment, user description, genre, and theme. Of the 6970 non-relevant tweets, 2384 (34.20%) were found to be entirely non-relevant, whereas the remainder were retweets with no additional context, conversations without context, or duplicated tweets from a user account that had since been suspended or was primarily being used for spam or unwanted solicitations.

**Table 1:** Supervised machine learning-based e-cigarette tweet classification categories (interrater reliability score for manual annotation).

Classification (Fleiss’ kappa)	Labels
Relevance^a^: Identifies tweets that are related to e-cigarettes (0.70)	Relevant
	Subcategory: retweet with no additional information
	Subcategory: original tweets that were part of a conversation and require greater context to be interpreted
	Subcategory: duplicated tweets from a user account that had since been suspended or was primarily being used for spam or unwanted solicitations
	Not relevant
Sentiment^b^: Indicates whether the stance in the tweet is positive, neutral, or negative towards e-cigarettes and users of e-cigarettes (0.65)	Positive
	Neutral
	Negative
User description^b^: Characterizes the sender of the tweet based on information gleaned from the user profile (0.66)	Celebrity
	Government
	Foundations or organizations
	Reputable news source
	Everyday people
	E-cigarette community movement
	Retailers
	Tobacco company
	Bots/hacked
Genre: Represents the format of the tweet (0.64)	Information
	First person e-cig use or intent
	Second/third person experience
	Personal opinion
	Marketing
	News/update
Theme: Refers to the topical domain of the content in the tweet (0.65)	Cessation
	Health and safety
	Underage usage
	Craving
	Other substances
	Illicit substance use in e-cigs
	Policy or government
	Parental use of e-cigs
	Advertisement/promotion
	Flavors

^a^Binary version of this category was created in addition to multiclass version for the purposes of the analysis.

^b^Categories were mutually exclusive and thus analyzed as multiclass.

### Tweet Classification Model Construction

Machine learning classification models were built for each of the five categories (relevance, user description, sentiment, genre, theme). In order to determine the best performing classifier model, several variations of classification techniques and word-grouping units (n-gram) were used (see Multimedia Appendices 3 and 4). In order for the classification model to distinguish relevant tweets from non-relevant tweets, the entire dataset of manually coded 17,098 tweets was used. All relevant tweets (10,128) were used to build classification models for the sentiment, user description, genre, and theme.

A mathematical representation of the tweet corpus was created based on the term frequency inverse document frequency transformation, which was preceded by the removal of stopwords and tokenization of text features established on count-based vectorization. For the final models, no attempt was made to reduce the feature space by using feature selection algorithms because exploratory analyses suggested no significant gain in performance and a potential decrease in predictive accuracy from implementing feature selection.

Three machine learning classification techniques—each based on alternative underlying statistical pattern recognition philosophies—were tested for each classifier: Naïve Bayes, k-Nearest Neighbors, and Support Vector Machines. In addition, word groupings (n-grams) ranging from unigrams to 5-grams were used to define the feature space for each classifier. The key attributes of the classification techniques used in this analysis are discussed in Multimedia Appendix 3 [30,31].

### Assessment of Model Performance and Sample Size

The preferred measure of predictive performance of the classification models implemented and reported in this analysis was the accuracy score, defined as the percentage of observations that were correctly classified in the validation dataset. This method was chosen due to its simplicity in interpretation, measure of overall effectiveness of a classification model [32], neutrality with respect to the weighting of false positive and false negatives, ease of comparison with other studies in the field, and suitability for multiclass variables.

The analysis implemented 10-fold cross validation as a means to avoid bias in the estimation of the accuracy score. This involved dividing the manually classified data into 10 groups, iteratively using combinations of nine distinct groups to fit the model and the remaining group to validate the performance of the model, and averaging the predictive performance score.

Performance scores were evaluated for each of the three classification techniques described above for feature spaces described by n-grams between unigrams and 5-grams. A total of 15 classification models were thus evaluated for each classifier (3 classification techniques x 5 n-gram specifications=15 classification models). Classifier refers to the categorical labels that were assigned during the manual annotation process that the machine classification models seek to correctly label. Classifiers resulting from mutually exclusive categories (ie, user description, sentiment) were analyzed as multiclass (could assume one of many class values) (see Table 1). Classifiers resulting from non-mutually exclusive categories—multilabel categories (eg, genre, theme) where one tweet could be assigned any one or more of many class values—were each assessed in terms of binary prediction performance for each constituent class (could assume only one of two class values). The relevance category was analyzed in the form of both binary and multiclass classifiers, given that data for subcategorization of the relevance category was also available. A total of 20 classifiers were assessed, each with 15 classification models.

Random accuracy baselines were computed for each binary and multiclass classifier to provide a point of performance comparison. The random baseline reflects how well a classification model would perform based on pure guesswork combined with knowledge of the true occurrence fraction of each class.

We assessed sample size adequacy by sequentially including 20%, 40%, 60%, 80%, and 100% of manually coded tweets and plotted a learning curve to visually examine where, if at all, the improvement in performance score begins to level off. As noted earlier, we also quantitatively assessed the feasibility of feature selection based on the chi-square method in improving efficiency and accuracy for a limited number of classifiers. This exploratory analysis concluded it was better to proceed without feature selection for the final models. The Python programming language version 2.7, in particular the Scikit Learn library version 0.15.1, was used for these analyses.

## Results

The predictive performance scores from the supervised machine learning–based analyses are presented in Table 2. The table reports accuracy scores for the best performing classification model, with consideration to the best performing classification technique and best word-grouping unit (n-gram) for each classifier. As a method of normalization, accuracy scores were additionally evaluated in terms of the percentage improvement achieved of the maximum possible improvement over the random baseline. A more complete table of results, which includes all classification models (all combinations of classification techniques and n-grams evaluated for each classifier), is included in Multimedia Appendix 4.

**Table 2:** Supervised machine learning-based e-cigarette tweet classification performance results.

Classifier labels	Best n-gram	Accuracy score	% achieved of possible improvement over random baseline
Relevance category^a^	1	0.75	57.25
Relevance	1	0.94	75.26
User description^a^	2	0.68	41.59
Sentiment^a^	2	0.76	46.05
News	1	0.93	52.26
Info	4	0.86	41.75
Personal experience	2	0.84	50.17
Second person	2	0.92	47.09
Personal opinion	2	0.79	48.93
Marketing	1	0.91	72.56
Cessation	1	0.95	58.43
Health and safety	1	0.90	56.29
Underage usage	1	0.97	58.92
Craving	2	0.97	58.43
Other substances^b^	1	0.99	49.42
Illicit substances	2	0.98	48.24
Policy or government	1	0.94	80.62
Parental use	1	0.99	54.40
Ad or promotion	1	0.89	72.69
Flavor	1	0.97	62.52

^a^Classifiers were multiclass. All other categories were binary.

^b^k-nearest neighbors (kNN) was the best performing classification technique; for all other cases, linear support vector machine (SVM) was best.

Predictive performance scores for classification models ranged between 0.68 and 0.99 indicating that the models correctly labeled the tweets with the appropriate variables between 68.40% and 99.34% of the time, and the percentage of maximum possible improvement over a random baseline that was achieved by the classification models ranged from 41.59% to 80.62%. The average performance score was 0.90 and the average improvement over a random baseline was 56.64%. Classifiers with the highest performance scores that also achieved the highest percentage of the maximum possible improvement over a random baseline were Policy/Government (performance: 0.94; % improvement: 80.62%), Relevance (performance: 0.94; % improvement: 75.26%), Ad or Promotion (performance: 0.89; % improvement: 72.69%), and Marketing (performance: 0.91; % improvement: 72.56%).

All classifiers performed best using the linear support vector algorithm with the exception of Other Substances, which performed best with the k-Nearest Neighbors algorithm. The most appropriate word-grouping unit (n-gram) was 1 for the majority of classifiers. Twelve classification models performed best with a unigram sequence, while seven performed best with a bigram sequence, only one performed best with a four-gram sequence, and none performed best with tri-gram or 5-gram sequences.

**Figure 1:**
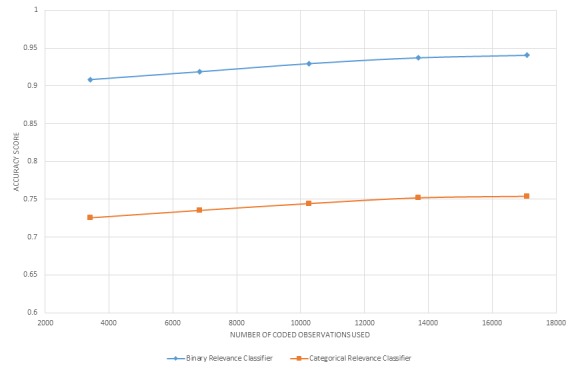
Learning curve for tweet relevance classification.

**Figure 2:**
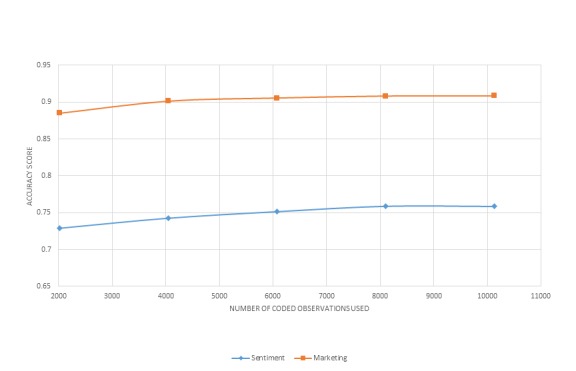
Learning curve for tweet topic classification.

Figures 1 and 2 display learning curves for a selection of classifiers. These curves indicate that performance continues to marginally increase with the size of the training dataset of manually annotated data but begins to level off at approximately 14,000 observations for relevance classification and approximately 8000 (relevant) observations for topic classification. However, even at low dataset sizes of 4000 observations, the performance characteristics are fairly sound. All classifiers, including those not displayed, followed this same pattern. Besides providing insight into how performance characteristics relate to sample size, these data suggest that an adequate training set of manually annotated data was deployed for the current analysis.

## Discussion

### Principal Findings

This work adds to the growing body of literature that highlights the importance of machine learning for large, language-based datasets of publicly available data. Using social media allows for exploration into conversations occurring outside of the traditional public health space, and machine learning provides an opportunity to keep abreast of these conversations in a more rapid fashion. The results of this study provide an example of the use of supervised machine learning methods to assess the vast social media landscape around e-cigarettes. This study used a five-category manually coded training set to train machine learning classification algorithms, thus categorizing e-cigarette-related content on Twitter with relative accuracy and detail. The findings provide insight into machine learning techniques that are most appropriate for assessing e-cigarette data around particular topics such as sentiment, speaker, and genre. The study provides a methodology that can be replicated to determine similar information about other public health-related trends and topics. Particular aspects of this methodology contribute to efforts to improve ethical use of Twitter data for public health, such as improving the signal-to-noise ratio [14]. For example, identification of the type of Twitter account allows information to be considered in context as opposed to considering all information disseminated via Twitter to be equal.

Of the classification techniques examined, linear support vector machines generally had the highest levels of predictive performance, which is consistent with the results of some previous text classification studies [25]. Unigrams were generally found to be the most successful word grouping for tweet classification, which is consistent with the short nature of tweets and their relative performance observed in previous studies [25,33].

The absolute performance scores from these models compare favorably to those reported in the literature for similar short text classification tasks [25,33]. For instance, Agarwal et al report classification accuracy ranging from 56.31% to 60.83% for a tertiary (positive, negative, neutral) sentiment analysis of manually annotated Twitter data [33], with a chance baseline of 33.33%, thus realizing from 34.47% to 41.25% of the total achievable improvement over the chance baseline. By comparison, our study realized 46.05% of the total achievable improvement over the chance baseline for a tertiary (positive, negative, neutral) sentiment classifier. For the 20 binary or multiclass variables considered in this study, this metric ranged between 41.59% and 80.61%.

Findings from learning curves assessing classification model performance by sample size of manually annotated data show that the sample size used in this study was sufficient to observe maximum performance of the classification models. Additionally, learning curve findings provide insight to future research to assess the optimal sample size of manually annotated data necessary to build such supervised machine learning algorithms. As Figueroa et al note, manual annotation (ie, manual content analysis) of data for supervised machine learning can be cumbersome; thus, knowledge such as that provided by this study can aid future researchers in making decisions related to optimization of the sample size for manually annotated training sets [27].

Further analyses of the rich dataset created as part of this work may contribute to the development of novel methods that could enhance the performance of automated surveillance tools. Unsupervised topic classification techniques could potentially be used in creative ways to improve the performance of the supervised learning classifier models. Machine learning–based image classification may add an additional dimension to automated surveillance tools assessing social media for insights and trends. The learning curve data developed for various combinations of algorithms and n-grams may be fitted with generalized mathematical functions that may potentially provide a basis for manual annotation sample size decision rules in other contexts.

Methods used in this study may find potential extensions in the development of automated social media infodemiology tools that could provide insight into the evolving social media landscape around e-cigarettes and other public health–related topics in real-time, thus providing valuable information for researchers, policy makers, and public health officials. Findings obtained from tools such as these could be used to inform interventions, policy, and communication strategies with up-to-date and time relevant information. Additionally, methods from this study can be used to support exploratory analysis and hypothesis generation on more nuanced aspects of a particular topic or to focus on a particular demographic or user group. Furthermore, the discoveries that this type of infodemiology yields could potentially be used to inform the public and test communication strategies to influence behavior in the interest of public health. Public health officials and researchers engaged in behavior change interventions, such as smoking cessation support, may even consider potentially developing custom applications based on the detection of and responses to particular tweet topics (eg, youth initiation of e-cigarette use).

Even though the Twitter analysis automation was successful, it is based on a manual content analysis, which may be subject to bias. Despite this, the coding scheme used for the manual content analysis was based on an existing scheme supported by the literature [25], and our coders had acceptable interrater reliability. For these reasons, we are confident in the validity of the results and the replicability of the methodology to further understand Twitter trends over time for e-cigarettes as well as other health topics. The implications of replication are far-reaching, especially as social media and other digital platforms continue to be a venue for unfiltered, real-time discourse.

### Conclusion

There is great potential for using new forms of data in social science and public health. As the world transitions to sending and receiving information online, social media outlets like Twitter hold the potential to uncover real-time snapshots of personal sentiment, knowledge, attitudes, and behavior that is not as accessible, at this scale, through any other offline platform. This medium is arguably one of the quickest and easiest means to identify trends or outbreaks and allow researchers, public health officials, and policy makers to respond in a collaborative way to inform the public about issues that can improve the quality and longevity of their lives. Despite the benefits of infodemiology, this nascent field presents unique ethical challenges as well as challenges that are inherent to the study of public health [21].

As we seek to understand the vast amount of data available via social media, social science and public health methodologies must adapt and use computational methodologies to enhance and extend research and practice. This study was successful in automating a complex five-category manual content analysis of e-cigarette–related Twitter content using machine learning techniques. The study detailed machine learning model specifications that provided the best accuracy for data related to e-cigarettes, as well as a replicable methodology to allow extension of these methods to additional topics. In the future, additional research will be needed to continue to enhance these methodologies and demonstrate their cost-effectiveness and feasibility as tools for intervention and real-time surveillance.

## Appendix

Multimedia Appendix 1 Tweet filter keywords.

Multimedia Appendix 2 Definitions of annotation categories.

Multimedia Appendix 3 Description and key attributes of machine learning classification.

Multimedia Appendix 4 Extended supervised machine learning-based e-cigarette tweet classification performances results.
