# Supplementary material for: Assessing Electronic Cigarette-Related Tweets for Sentiment and Content Using Supervised Machine Learning
Source: J Med Internet Res. 2015 Aug 25;17(8):e208. doi: 10.2196/jmir.4392 (PMC4642404; doi:10.2196/jmir.4392)
Supplement: Multimedia Appendix 1 [file jmir_v17i8e208_app1.pdf]

Multimedia Appendix 1. Tweet filter keywords.

Vaping
Vape
Vaper
Vapers
Vapin
Vaped
Evape
Vaporing
e-cig*[a]
ecig*[a]
e-pen
epen
e-juice
ejuice
e-liquid
eliquid
cloud chasing
cloudchasing
deeming AND regulation
deeming AND FDA
deemed AND FDA
Deem*[a] and FDA

[a]Word stem used to identify any word beginning with these characters.
