# Supplementary material for: Assessing Electronic Cigarette-Related Tweets for Sentiment and Content Using Supervised Machine Learning
Source: J Med Internet Res. 2015 Aug 25;17(8):e208. doi: 10.2196/jmir.4392 (PMC4642404; doi:10.2196/jmir.4392)
Supplement: Multimedia Appendix 3 [file jmir_v17i8e208_app3.pdf]

Multimedia Appendix 3. Description and key attributes of machine learning classification techniques.

Classification Technique	Description
Naïve Bayes	The Naïve Bayes approach, which is frequently used as a baseline classification technique in many text classification analyses, assumes knowledge of the underlying class-conditional probability distribution functions of the feature vectors. In machine learning applications, the parameters of these probability distribution functions will usually be unknown and must be estimated from the annotated training dataset [31]. The Naïve Bayes algorithm is an example of an approach typically based on a parametric estimation of the distribution function and assumes that individual features are all conditionally independent of one another for a given class.
k-Nearest Neighbors	The k-Nearest Neighbors algorithm is conceptually one of the simplest classification techniques and is based on ‘judging a person by the company he keeps’ [33]. This method estimates the underlying class-conditional probability distribution functions of the feature vectors non-parametrically. The most commonly used metrics in measuring the distance of an unclassified sample from a classified prototype (in the training dataset) are Euclidean distance and cosine angle distance.
Support Vector Machines	The support vector machines algorithm is widely reported as being amongst the most successful text classification methods particularly for short documents. This method does not require explicit computation of the probability distribution functions of the feature vectors but instead develops decision rules that use the data to estimate the decision boundaries directly [34]. Linear support vector machines work on the idea of estimating a hyperplane that separates two classes of observations. The model’s support vector estimations may be performed using optimization methods drawn from a wide family of techniques, including interior point methods, sequential minimal optimization and, in special cases, gradient descent algorithms. The support vector machine algorithm is mostly designed for binary variables; multiclass variables are most often classified by constructing multiple binary variables.
