# Supplementary material for: Assessing Electronic Cigarette-Related Tweets for Sentiment and Content Using Supervised Machine Learning
Source: J Med Internet Res. 2015 Aug 25;17(8):e208. doi: 10.2196/jmir.4392 (PMC4642404; doi:10.2196/jmir.4392)
Supplement: Multimedia Appendix 4 [file jmir_v17i8e208_app4.pdf]

**Multimedia Appendix 4.** Extended supervised machine learning-based e-cigarette tweet classification performance results.

| Category Name | Type | Best Classifier | Best n-gram | Accuracy Score | Chance Baseline Score | Improvement Over Baseline | % Achieved of Possible Improvement Over Baseline |
|---|---|---|---|---|---|---|---|
| Relevance Category[a] | Multinomial | LinearSVC | 1 | 0.75 | 0.42 | 0.33 | 57.25% |
| Relevance | Binomial | LinearSVC | 1 | 0.94 | 0.76 | 0.18 | 75.26% |
| User Description[a] | Multinomial | LinearSVC | 2 | 0.68 | 0.46 | 0.22 | 41.59% |
| Sentiment[a] | Multinomial | LinearSVC | 2 | 0.76 | 0.55 | 0.21 | 46.05% |
| News | Binomial | LinearSVC | 1 | 0.93 | 0.85 | 0.08 | 52.26% |
| Info | Binomial | LinearSVC | 4 | 0.86 | 0.75 | 0.10 | 41.75% |
| Personal Experience | Binomial | LinearSVC | 2 | 0.84 | 0.68 | 0.16 | 50.17% |
| Second Person | Binomial | LinearSVC | 2 | 0.92 | 0.85 | 0.07 | 47.09% |
| Personal Opinion | Binomial | LinearSVC | 2 | 0.79 | 0.60 | 0.20 | 48.93% |
| Marketing | Binomial | LinearSVC | 1 | 0.91 | 0.67 | 0.24 | 72.56% |
| Cessation | Binomial | LinearSVC | 1 | 0.95 | 0.88 | 0.07 | 58.43% |
| Health and Safety | Binomial | LinearSVC | 1 | 0.90 | 0.77 | 0.13 | 56.29% |
| Underage Usage | Binomial | LinearSVC | 1 | 0.97 | 0.92 | 0.05 | 58.92% |
| Emotional Response | Binomial | LinearSVC | 2 | 0.85 | 0.73 | 0.12 | 44.44% |
| Craving | Binomial | LinearSVC | 2 | 0.97 | 0.93 | 0.04 | 56.03% |
| Other Substances | Binomial | KNeighborsClassifier | 1 | 0.99 | 0.98 | 0.01 | 49.42% |
| Illicit Substances | Binomial | LinearSVC | 2 | 0.98 | 0.97 | 0.02 | 48.24% |
| Policy or Government | Binomial | LinearSVC | 1 | 0.94 | 0.68 | 0.26 | 80.62% |
| Parental Use | Binomial | LinearSVC | 1 | 0.99 | 0.99 | 0.01 | 54.40% |
| Normalization | Binomial | LinearSVC | 3 | 0.75 | 0.63 | 0.12 | 32.91% |
| Ad or Promotion | Binomial | LinearSVC | 1 | 0.89 | 0.61 | 0.28 | 72.69% |
| Flavor | Binomial | LinearSVC | 1 | 0.97 | 0.91 | 0.05 | 62.52% |

[a]Classifiers were multiclass. All other categories were binary.
